# *Legionella pneumophila* temporally regulates the activity of ADP/ATP translocases by reversible ADP‐ribosylation

**DOI:** 10.1002/mlf2.12014

**Published:** 2022-03-30

**Authors:** Jiaqi Fu, Pengwei Li, Hongxin Guan, Dan Huang, Lei Song, Songying Ouyang, Zhao‐Qing Luo

**Affiliations:** ^1^ Department of Biological Sciences, Purdue Institute for Inflammation, Immunology and Infectious Disease Purdue University West Lafayette Indiana USA; ^2^ Provincial University Key Laboratory of Cellular Stress Response and Metabolic Regulation, The Key Laboratory of Innate Immune Biology of Fujian Province, Biomedical Research Center of South China, Key Laboratory of OptoElectronic Science and Technology for Medicine of the Ministry of Education, College of Life Sciences Fujian Normal University Fuzhou China; ^3^ Key Laboratory of Organ Regeneration and Transplantation of the Ministry of Education, State Key Laboratory of Zoonotic Diseases, Department of Respiratory Medicine, Center for Pathogen Biology and Infectious Diseases The First Hospital of Jilin University Changchun China

**Keywords:** energy metabolism, metaeffector, mitochondria, type IV secretion

## Abstract

The mitochondrion is an important signaling hub that governs diverse cellular functions, including metabolism, energy production, and immunity. Among the hundreds of effectors translocated into host cells by the Dot/Icm system of *Legionella pneumophila*, several are targeted to mitochondria but the function of most of them remains elusive. Our recent study found that the effector Ceg3 inhibits the activity of ADP/ATP translocases (ANTs) by ADP‐ribosylation (ADPR). Here, we show that the effect of Ceg3 is antagonized by Larg1, an effector encoded by *lpg0081*, a gene that is situated next to *ceg3*. Larg1 functions to reverse Ceg3‐mediated ADPR of ANTs by cleaving the N‐glycosidic bond between the ADPR moiety and the modified arginine residues in ANTs, leading to restoration of their activity in ADP/ATP exchange. Structural analysis of Larg1 and its complex with ADPR reveals that this ADPR glycohydrolase harbors a unique macrodomain that catalyzes the removal of ADPR modification on ANTs. Our results also demonstrate that together with Ceg3, Larg1 imposes temporal regulation of the activity of ANTs by reversible ADPR during *L. pneumophila* infection.

## Impact statement

Mitochondrion is not only the energy factory of eukaryotic cells, but also a signaling hub important for cellular activities such as immunity and cell death. It is thus a common target for infectious agents. An earlier study found that the *Legionella pneumophila* effector Ceg3 inhibits host energy metabolism by attacking ADP/ATP translocases by ADP‐ribosylation. This study reveals that such inhibition is regulated by Larg1, which functions as an ADPR glycohydrolase to antagonize the activity of Ceg3 at later phase of the infection cycle, highlighting the importance of temporal regulation of host activity in interactions between *L. pneumophila* and its hosts.

## INTRODUCTION

To survive and replicate in host cells, *Legionella pneumophila* has evolved a number of effective strategies to modulate host pathways to create an intracellular niche termed *Legionella*‐containing vacuole (LCV) that is permissive for bacterial growth. One major factor essential for the creation of the LCV is the Dot/Icm type IV secretion system, which injects over 330 protein substrates into host cells^1^, ^2^, ^3^. These effectors have been shown to modulate various signaling pathways by specifically targeting distinct organelles to promote the biogenesis of the LCV^4^. For example, a number of effectors target the endoplasmic reticulum (ER) to facilitate the conversion of the membranes of the nascent phagosome into an ER‐like organelle^5^, ^6^, ^7^. As expected, effectors that interfere with host gene expression are targeted to the nuclei of infected cells^8^, ^9^.

Mitochondrion plays an essential role in cell physiology as the center for energy metabolism, a guard for cell integrity, and an important signaling knot in immunity^10^. A few years after *L. pneumophila* was recognized as a pathogen, Horwitz et al.^11^ reported that close to 30% of LCVs in macrophages are associated with mitochondria within 15 min after bacterial internalization and such rates of association increased to larger than 65% when infection time was extended to 1 h. Late studies revealed that such association is virulence dependent as vacuoles harboring mutants lacking a functional Dot/Icm transporter are devoid of this organelle^12^. Consistent with this Dot/Icm‐dependent mitochondria recruitment, a number of its substrates have been found to specifically target this organelle, including MitF, LegS2, LncP, Lpg1625, Lpg0898, Lpg2444, and Ceg3^13^. Among these, MitF modulates mitochondrial dynamics to promote a Warburg‐like effect in infected macrophages, which favors bacterial replication^14^. LegS2 disrupts host sphingolipid metabolism to inhibit autophagy by functioning as a sphingosine 1‐phosphate lyase^3^, ^15^. Ceg3 recently was found to localize to the mitochondria where it targets ADP/ATP translocases (ANTs) by mono‐ADP‐ribosylation (ADPR), resulting in the inhibition of its ADP/ATP exchange activity^13^. The function of LncP, Lpg1625, Lpg0898, and Lpg2444 remains unknown.

It is well‐established that bacterial pathogens utilize different sets of virulence factors to modulate host function at different phases of infection^16^. For instance, *Listeria monocytogene* employs InlA and InlB for host cell entry and later utilizes LLO, PLCs, and ActA to facilitate its escape from the phagosome and subsequent cell‐cell spread^17^. In some cases, temporal regulation of virulence is achieved by virulence factors that directly impact the activity of each other^18^. In this regard, recent studies have revealed that *L. pneumophila* has evolved sophisticated and diverse mechanisms to regulate effector activity by metaeffectors^18^, ^19^. The first described mechanism is the targeting of SidH for proteasome degradation by LubX, which itself is a ubiquitin E3 ligase^20^. The second mechanism is to inactivate the target effector by posttranslational modification (PTM), which is exemplified by the inhibition of the SidE family of phosphoribosyl ubiquitin ligases by SidJ‐mediated glutamylation^21^, ^22^, ^23^. The third is by the formation of protein complexes between the effectors, which is represented by MesI that targets SidI^24^, ^25^ and by Lpg2149 that neutralizes MavC and MvcA^26^, ^27^. The most commonly used mechanism is the removal of the modification that is catalyzed by a different effector. This mode of action has been documented for several effector pairs, including AMPylation by SidM and SidD^28^, phosphorycholination by AnkX and Lem3^29^, ^30^, phosphoribosyl ubiquitination by SidEs and DupA/DupB^31^, ^32^, and the atypical transglutamination‐induced monoubiquitination by MavC and MvcA^33^, ^34^.

ADP/ATP carriers (ANTs) are responsible for transporting ATP into the cytosol from the matrix of the mitochondria to replenish cells with energy currency. Our earlier study found that the *L. pneumophila* effector Ceg3 inhibits the activity of ANTs by catalyzing ADPR on one arginine residue in a region important for their function^13^. Because the integrity of infected cells is essential for productive intracellular bacterial replication and prolonged inhibition of ANTs activity is detrimental to cell viability, we envisioned that the bacterium may erase the modification and return ANTs to their active form. This notion was supported by the observation that ADPR of ANTs peaks at 10 h post uptake and the modification becomes less abundant as the infection proceeds^13^, suggesting active removal of the modification by factors from the pathogen or the host. In this study, we present evidence to show that Lpg0081 (designated as Larg1 for *Legionella* ADP‐ribose glycohydrolase 1), a protein encoded by a gene adjacent to *ceg3*, functions with the ADP‐ribosyltransferase to temporally regulate the activity of ANTs. Our biochemical and structural analyses reveal that Larg1 harbors a unique macrodomain that functions to remove the ADPR moiety from modified ANTs, and it works together with Ceg3 to temporally regulate the activity of these translocases during *L. pneumophila* infection.

## RESULTS

### The effector Larg1 (Lpg0081) suppresses the yeast toxicity induced by Ceg3

**Figure 1:**
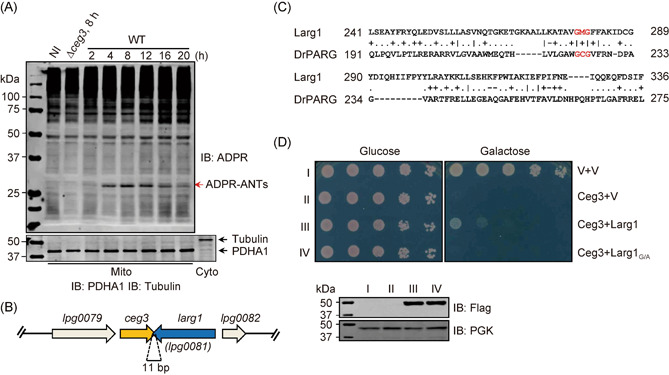
Larg1 inhibits the yeast toxicity of Ceg3 via its putative macrodomain. (A) ADP‐ribosylation (ADPR) of ANTs by Ceg3 in cells infected with *Legionella pneumophila* displays infection phase‐dependent fluctuation. Mitochondrial fractions of HEK293T cells infected with wild‐type *L. pneumophila* were isolated at the indicated time points after bacterial uptake and the presence of modified ANTs was probed by immunoblotting with ADPR‐specific antibodies. Uninfected (NI) samples and cells infected with the ∆*ceg3* mutant for 8 h were included as controls. Bands representing modified ANTs were indicated by a red arrow (upper panel). The effectiveness of cell fraction was examined by probing the mitochondrial resident protein PDHA1 with a cytosolic fraction sample as a control (lower panel). (B) The organization of genes *ceg3* and *larg1(lpg0081)* on the chromosome of *L. pneumophila* strain Philadelphia 1. (C) Larg1 harbors a putative macrodomain. HHpred analysis reveals the presence of a putative macrodomain resembling the one found in the poly(ADP‐ribose)glycohydrolase from *Deinococcus radiodurans* (DrPARG). Symbols indicate the quality of the column–column match: “I” very good, “+” good, “.” neutral, “−” bad. (D) Larg1 suppresses the yeast toxicity of Ceg3. A yeast strain expressing Ceg3 by the galactose‐inducible promotor was transformed with plasmids carrying Flag‐tagged Larg1 or its G/A mutant driven by a constitutive promoter. Serially (5‐fold) diluted cells were spotted on the indicated media. Images were acquired after 3‐day incubation at 28°C (upper panel). The expression of Larg1 or its mutant was probed with the Flag‐specific antibody. The PGK (3‐phosphoglycerate kinase) was probed as a loading control (lower panel). ANT, ADP/ATP translocase; V, vector; WT, wild type.

In our experiments to determine Ceg3‐induced ADPR of ANTs by wild‐type *L. pneumophila* at different phases after bacterial entry, we consistently observed that the amounts of ADP‐ribosylated ANTs began to decrease after reaching the maximum around 12 h post bacterial uptake (Figure 1A). This phenomenon is resembling SidM‐mediated recruitment of its target Rab1 onto the LCV^30^, suggesting the existence of an effector that functions to remove the modification on the ATP/ADP carriers. Because most of the effectors with opposite biochemical activity in regulating their host target are coded for by genes that are in close proximity on *L. pneumophila* chromosome^19^, we inspected the *ceg3* locus to search for the candidate gene. Upstream of *ceg3* is *lpg0079*, which is predicted to code for a 2‐polyprenyl‐6‐methoxyphenol hydroxylase involved in ubiquinone biosynthesis^35^, and its high‐level similarity to a metabolic enzyme makes it less likely to be an effector. In contrast, Lpg0081 encoded by the gene downstream of *ceg3* has been identified as a Dot/Icm effector^36^.

An earlier study has found that Larg1 suppresses the yeast toxicity of Ceg3^19^, suggesting that it may function to antagonize the ADP‐ribosyltransferase activity. On the *L. pneumophila* chromosome, the *ceg3* and *lpg0081* genes overlap by 11‐bp and are transcribed convergently (Figure 1B). Furthermore, bioinformatic analysis by HHpred^37^ revealed that Lpg0081 contains a putative macrodomain that is similar to an ADP‐ribose‐bound poly(ADP‐ribose) glycohydrolase from the radioresistant bacterium *Deinococcus radiodurans* (DrPARG)^38^ (Figure 1C). The Gly residues at positions 279 and 281 in Lpg0081 are conserved with those in DrPARG, which have been shown to be important for binding ADP‐ribose via hydrogen bonds^38^ (Figure 1C). Because of its potential ADP‐ribose glycohydrolase activity, we designated Lpg0081 as Larg1 (*Legionella* ADP‐ribose glycohydrolase 1). Similar to earlier observations^19^, Larg1 effectively suppressed the yeast toxicity of Ceg3 (Figure 1D). Furthermore, alanine substitution of Gly_279_ and Gly_281_ (designated as Larg1_G/A_) abolished its ability to suppress the yeast toxicity of Ceg3 without affecting protein stability (Figure 1D). Thus, the suppression of the yeast toxicity of Ceg3 by Larg1 likely is mediated by its ability to remove ADP‐ribose from ANTs via activity conferred by the putative macrodomain.

### Larg1 is not essential for intracellular bacterial replication

The ability of Larg1 to counteract the activity of Ceg3 suggests that it is a metaeffector for the ADP‐ribosytransferase. Several metaeffectors, including SidJ^23^ and MseI^24^, ^25^, ^39^, have been shown to be required for optimal virulence of *L. pneumophila;* we, thus, investigated the role of Larg1 in intracellular replication by constructing a ∆*larg1* mutant and examined its ability to replicate in macrophages and the protozoan host *Dictyostelium discoideum*, respectively. Similar to Ceg3 and many other Dot/Icm substrates, our results showed that the mutant grew as proficiently as the wild‐type strain (Figure S1), indicating that Larg1 is not essential for optimal intracellular bacterial replication in these commonly used infection models.

### Larg1 removes the ADPR modification on ANTs mediated by Ceg3

**Figure 2:**
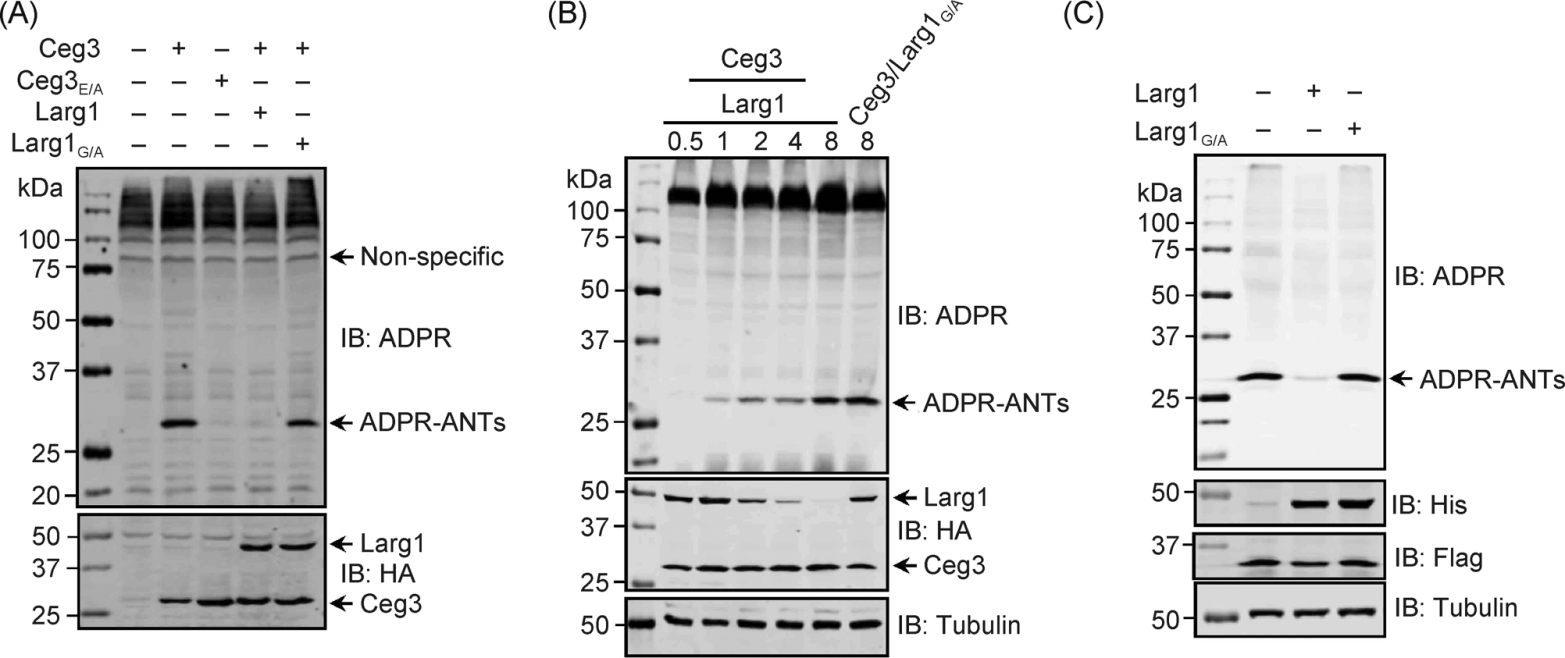
Larg1 functions to remove the modification from ADP‐ribosylated ANTs induced by Ceg3. (A) Larg1 inhibits Ceg3‐mediated ADP‐ribosylation (ADPR) of ANTs in mammalian cells. HEK293T cells were transfected to express the indicated proteins. The expression of the relevant proteins was probed with the HA antibody. ADP‐ribosylated endogenous ANTs were detected by immunoblotting with ADPR‐specific antibodies. Larg1_G/A_ is the mutant in which Gly_279_ and Gly_281_ have been replaced with alanine. (B) Dose‐dependent inhibition of Ceg3‐induced ANTs ribosylation by Larg1. Lysates of HEK293T cells transfected with plasmids that direct the expression of HA‐Ceg3 and HA‐Larg1 at the indicated ratios were probed with ADPR‐specific antibodies to detect ADPR‐ANTs. The expression of Ceg3 and Larg1 was detected using the HA‐specific antibody. Tubulin was probed as a loading control. (C) Larg1 removes ADPR on ANTs in a cell‐free reaction. Recombinant His_6_‐tagged Larg1 or Larg1_G/A_ were added to lysates of cells expressing Ceg3 and the reactions were allowed to proceed for 1 h at 37°C. Samples resolved by SDS‐PAGE were probed by the ADPR‐specific antibody to determine the ADPR level of ANTs. Note that Larg1 but not Larg1_G/A_ almost completely removed the ADP‐ribose moiety from ANTs. ANT, ADP/ATP translocase; HA, hemagglutinin; SDS‐PAGE, sodium dodecyl sulfate polyacrylamide gel electrophoresis.

To further determine the mechanism of the potential interference of Larg1 on Ceg3 activity, we coexpressed these two proteins in HEK293T cells. Consistent with results from our recent study^13^, Ceg3 but not Ceg3_E/A_ induced ADPR of endogenous ANTs. Importantly, the band representing ADP‐ribosylated ANTs almost disappeared in samples coexpressing Larg1 but not the Larg1_G/A_ mutant (Figure 2A), indicating that Larg1 interferes with the activity of Ceg3 in mammalian cells in a manner that requires the putative macrodomain. The inhibition of Ceg3 activity by Larg1 was dose‐dependent (Figure 2B). Under our experimental conditions, at a ratio (the plasmid amounts used for transfection between Ceg3 and Larg1) of ~4:1, the decrease of modified ANTs became apparent.

To further determine the mechanism underlying the demodification of ANTs by Larg1, we incubated cell lysates containing ADP‐ribosylated ANTs with recombinant His_6_‐Larg1 protein or His_6_‐Larg1_G/A_ at 37°C for 1 h. ADP‐ribosylated ANTs became almost undetectable in reactions receiving wild‐type Larg1 (Figure 2C). Consistent with its inability to rescue the Ceg3‐induced yeast toxicity, Larg1_G/A_ was unable to demodify ADPR‐ANTs (Figure 2C), suggesting that the predicted macrodomain is critical for the function of Larg1. Thus, Larg1 antagonizes the activity of Ceg3 by direct removal of the ADP‐ribosylation modification on ANTs.

### Larg1 partially localizes to the mitochondria

**Figure 3:**
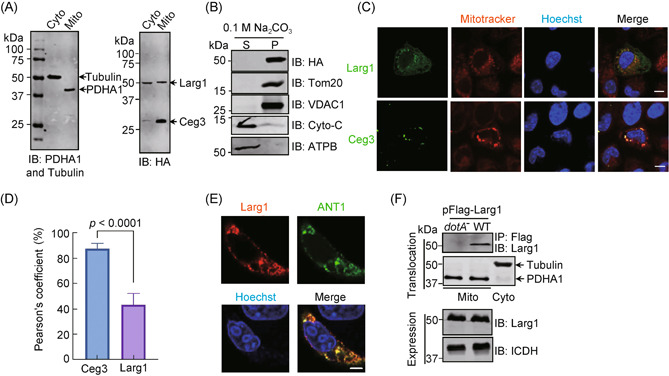
Larg1 is localized to the mitochondria. (A) A portion of Larg1 fractionates with mitochondria. The cytosol and mitochondrial fractions of HEK293T cells transfected to express HA‐Ceg3 and HA‐Larg1 were probed with the HA‐specific antibody. PDHA1 and tubulin were probed as markers for the mitochondrial and the cytosol fraction, respectively. (B) The association of Larg1 with the mitochondria is not peripheral. Mitochondria isolated from HEK293T cells expressing HA‐Larg1 were subjected to extraction with a high pH buffer (0.1 M Na_2_CO_3_, pH 11) for 30 min. Relevant proteins in soluble (S) and pellet (P) fractions separated by high‐speed centrifugation were probed with the indicated antibodies. (C,D) Larg1 colocalizes with the mitochondrion. HeLa cells transfected to express HA‐Larg1 for 18 h were treated with Mitotracker Red for 30 min before immunostaining with the HA‐specific antibody (green) (upper panels). Cells transfected to express Flag‐Ceg3 were used as control (lower panels). In each case, images were acquired with a Zeiss LSM 880 confocal microscope after staining the nuclei with Hoechst. Bar, 5 μm (C). The colocalization of Larg1 and Ceg3 with mitochondria was quantitated by determining the Pearson's correlation coefficient with Image J. Error bars: standard error of the mean (SEM). Statistical analysis was determined by a two‐tailed *t* test (D). (E) Larg1 colocalizes with ANT1. Cells transfected to express HA‐Larg1 and mCherry‐ANT1 were stained with the HA antibody. Images were acquired with a Zeiss LSM 880 confocal microscope after staining the nuclei with Hoechst. Bar, 5 μm. (F) Larg1 is targeted to mitochondria in cells infected with *Legionella pneumophila* by the Dot/Icm system. HEK293T cells expressing the FcγII receptor were infected with opsonized bacteria expressing Flag‐Larg1 at an MOI of 100 for 2 h. Lysates of isolated mitochondria were subjected to immunoprecipitation with beads coated with the Flag antibody, and precipitates were probed by the Larg1‐specific antibody (top). The quality of cell fractionation was evaluated by detecting PDHA1 and tubulin. A cytosolic fraction sample was included as an additional control (middle). The expression of Larg1 in bacteria was detected with the Larg1 antibody, and ICDH was probed as a loading control (bottom). ICDH, isocitrate dehydrogenase; MOI, multiplicity of infection.

ANTs are carrier proteins that function to facilitate ADP and ATP exchange across the mitochondrial inner membranes^40^. To remove the ADPR moiety from ANTs, Larg1 needs to be targeted to the mitochondria. We next examined whether Larg1 is associated with this organelle by coexpressing hemagglutinin (HA)‐Ceg3 and HA‐Larg1 in HEK293T cells. The cytosol and mitochondrial fractions were obtained by cell fractionation. Immunoblotting with antibodies specific to tubulin and PDHA1 (the subunit α1 of pyruvate dehydrogenase E1, a mitochondrial resident protein)^41^, respectively, indicated that separation of the mitochondria was successful. When these samples were probed with the HA‐specific antibody, we found that Ceg3 was detected in the mitochondrial fraction. Approximately 50% of HA‐Larg1 cofractionated with PDHA1, suggesting that Larg1 is partially targeted to the mitochondria, differing from Ceg3, which is almost completely targeted to this organelle (Figure 3A).

We next examined how Larg1 is associated with mitochondrial membranes. Mitochondria isolated from cells expressing HA‐Larg1 were treated with a high pH carbonate buffer (0.1 M Na_2_CO_3_, pH 11), then integral and peripheral mitochondrial membrane proteins were separated by centrifugation. HA‐Larg1 was cofractionated with two integral mitochondrial membrane proteins, Tom20 and VDAC1, but not with Cyto‐c or ATPB, two peripheral membrane proteins of this organelle (Figure 3B). Thus, a portion of Larg1 is stably associated with mitochondria, where it likely reaches the inner membranes to engage ADPR‐ANTs.

We further analyzed its cellular localization by immunostaining in cells transfected to express HA‐Larg1. Consistent with results from cell fractionation data, the staining signals of HA‐Larg1 overlapped partially with those of a Mitotracker dye while Ceg3 displayed more specific localization to mitochondria (Figure 3C,D). Similar colocalization between Larg1 and ANT1 was also detected (Figure 3E). These results further indicate that at least a portion of Larg1 enters the mitochondria. Finally, we also detected Larg1 in mitochondrial fractions of cells infected with a wild‐type *L. pneumophila* strain overexpressing Flag‐Larg1 but not with a mutant lacking a functional Dot/Icm transporter (Figure 3F), suggesting that Larg1 reaches mitochondria once being injected into host cells.

### Larg1 cleaves the N‐glycosidic bond between the ADPR moiety and ANTs

**Figure 4:**
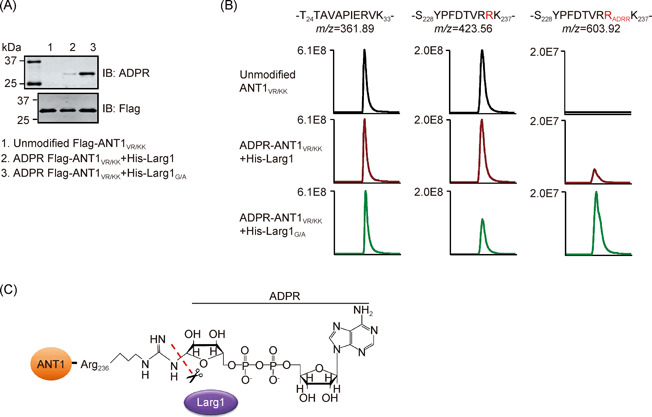
Larg1 cleaves the N‐glycosidic bond that links ADPR‐ANTs. (A) Larg1 removes the ADPR moiety from modified ANT1_V227K/R237K_(ANT1_VR/KK_). ADP‐ribosylated ANT1_V227K/R237K_ purified by immunoprecipitation from lysates of cells transfected to express Flag‐ANT1_V227K/R237K_ and HA‐Ceg3 was incubated with His_6_‐Larg1 or His_6_‐Larg1_G/A_ at 37°C for 1 h. Samples resolved by SDS‐PAGE were probed with antibodies specific to ADPR and Flag, respectively. Similarly purified unmodified Flag‐ANT1_V227K/R237K_ coexpressed with HA‐Ceg3_E/A_ was used as control. (B) ADP‐ribosylated ANT1_V227K/R237K_ is restored to its original form by Larg1. Three differently treated ANT1 samples were analyzed by mass spectrometry. Extracted ion chromatograms of peptides—S_228_YPFDTVRRK_237_—with ADPR (*m*/*z* = 603.92) and without (*m*/*z* = 423.56) modification are shown. ANT1_V227K/R237K_ peptide—T_24_TAVAPIERVK_33_—was used as an internal control. (C) A diagram depicting the site of cleavage by Larg1. The side chain of Arg236 in ANT1 was linked to the ADPR moiety by an N‐glycosidic bond, which was hydrolyzed by Larg1.

To more precisely determine the removal of the ADPR moiety from modified ANTs by Larg1, we incubated ADP‐ribosylated ANT1_V227K/R237K_ with recombinant Larg1 and probed the modification by immunoblotting and observed a clear reversal of the modification (Figure 4A). In theory, such cleavage could occur either at the N‐glycosidic bond as ARH1 does^42^ or at the phosphoanhydride bond similar to the ADPR pyrophosphatase NUDT^43^. We then probed the cleavage mechanism by analyzing ADPR‐ANT1_V227K/R237K_ that has been treated by Larg1 or its enzymatically inactive mutant with mass spectrometry (MS). Compared to samples treated with the inactive Larg1_G/A_ mutant, the intensity of the ADP‐ribosylated peptide—S_228_YPFDTVR_ADPR_RK_237_—detected by liquid chromatography with tandem mass spectrometry (LC‐MS/MS) drastically decreased in samples that had been incubated with wild‐type protein (Figure 4B). This decrease was accompanied by an increase in signals representing unmodified peptide—S_228_YPFDTVRRK_237_—(Figure 4B), indicating that modified ANT1_V227K/R237K_ was restored to its original form after reacting with Larg1. Thus, Larg1 specifically cleaves the N‐glycosidic bond between the ADPR moiety and the modified arginine residues in ANTs.

### A unique macrodomain in Larg1 engages the ADPR moiety for its removal

**Figure 5:**
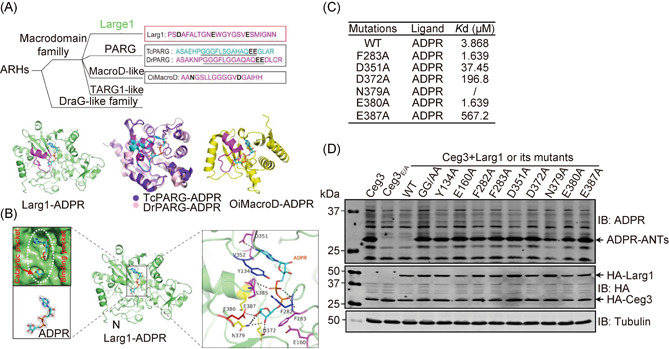
Larg1 recognizes ADPR by a unique macrodomain. (A) Classification of macrodomains associated with bacterial ADP‐ribosylhydrolases. The sequences involved in the formation of the catalytic loop of a typical member of macrodomain families are shown in magenta (Larg1, DrPARG, and OiMacroD) or cyan (TcPARG) letters, respectively. The residues critical for the catalytic activity of these hydrolases are shown in black letters (upper panel). The structures of the complex formed by ADPR with Larg1, TcPARG, DrPARG, or OiMacroD are shown as green purple, pink, and yellow, respectively (lower panels). (B) Interactions between Larg1 and ADPR. Left panel: The ADPR binding pocket of Larg1 is indicated by a dashed circle, and the catalytic pocket is indicated by an arrow (top portion). The 2Fo‐Fc map of the ADPR is contoured at 1.2 σ (bottom portion). Right panel: Residues of Larg1 involved in the formation of the pocket for ADPR, direct interactions between the side chains of these residues (shown in different colors) and ADPR are indicated by dashed lines. (C) Identification of residues important for the binding of Larg1 to ADPR. The binding affinity was evaluated using isothermal titration calorimetry. The dissociation constant (*K*d) was calculated by Launch NanoAnalyze software. Data shown are one representative from three experiments with similar results. (D) Larg1 residues important for recognizing ADP‐ribose are required for its enzymatic activity. Lysates of HEK293T cells transfected to coexpress HA‐Ceg3 and HA‐Larg1 or its mutants were resolved by SDS‐PAGE and probed with ADPR‐specific antibodies for ADPR‐ANTs and HA‐specific antibodies for the level of relevant proteins. Tubulin was probed as a loading control.

To better understand the mechanism of ADPR removal from modified ANTs by Larg1, we crystallized and solved the structure of the Larg1‐ADPR complex to a resolution of 2.32 Å, which reveals that the protein is composed of a central nine‐stranded mixed β‐sheet sandwiched by six α‐helices, a feature that is very similar to that found in other α/β macrodomains^44^. We compared the crystal structure of Larg1 with entries in the PDB using the Dali server (http://ekhidna.biocenter.helsinki.fi/dali_server/). The best hits were MavL (PDB: 6OMI) and TcPARG. MavL is a Dot/Icm effector of *L. pneumophila* ^2^, which may possess ADP‐ribosylhydrolase activity but has not been well studied. TcPARG has been reported as a poly(ADP‐ribose) glycohydrolase (PARG) from *Thermomonospora curvata* ^45^. The root mean square deviation (RMSD) and the *Z*‐score between Larg1 and TcPARG by Dali are 3.1 and 9.5, respectively. Furthermore, the catalytic loop of Larg1 is formed by an element consisting of 19‐residue (‐S_371_DAFALTGNEWGYGSVES_388_‐) that is structurally similar to that of bacterial PARGs, such as TcPARG, DrPARG, and the MacroD‐like hydrolase OiMacroD^46^ despite these proteins not sharing significant sequence similarity in this region (Figure 5A). The structure of Larg1 in its complex with ADPR reveals that the three conserved residues Asp372, Glu380, and Glu387, likely function as its catalytic site.

To gain insights into the molecular recognition mechanism of the hydrolase toward its substrate, we investigated the binding pocket for ADPR in the Larg1‐ADPR complex and found that the ADPR moiety is situated in the macrodomain pocket where the binding is coordinated by interactions mediated by several pairs of hydrogen bonds provided by surrounding amino acids with residues Tyr134 and Phe283 forming a “lid‐shape” element on the top of the pocket. The side chain of Asp351 and the main chain of Val352 contact the hydrogen atom and nitrogen atom of the pyrimidine ring in the adenine moiety via direct hydrogen bonding, respectively. The oxygen atoms of the pyrophosphate in ADPR make contact with adjacent residues via hydrogen bonding with hydrogen atoms in the main chain of Gly279, Gly281, Phe282, Phe283 of α10 and Ser385 of α13. Additionally, the ribose moiety of ADPR forms hydrogen bonds with Asp372, Gln379, and Glu387, and the three residues form a catalytic pocket and facilitate positioning ribose moiety of ADPR (Figure 5B). The negatively charged Glu380 located in the catalytic loop attacks the N‐glycosidic bond between Arg_236_ of ANT1 and ADPR (Figure 5B).

To validate the importance of residues predicted to be critical for ADPR recognition by Larg1 in the structure, we created mutants by introducing Ala substitution in Phe283, Asp351, Asp372, Asn379, Glu380, and Glu387, respectively. We first examined the binding of these mutants to ADPR using isothermal titration calorimetry (ITC). In the structure, Asp351 and Val352 function to stabilize the adenine. Consistently, the D351A mutant displayed a 10‐fold reduction in its binding affinity to ADPR. Similarly, mutants D372A, N379A, and E387A are involved in the formation of a catalytic pocket, and each has severely lost the ability to bind ADPR (binding between the N379A mutant and ADPR was undetectable). In contrast, the E380A mutation did not detectably impact the interaction, nor did the mutation in Phe283, the residue involved in the formation of the “lid” (Figure 5C).

We also examined the role of these residues in the hydrolase activity of Larg1 by mutating Tyr134, Glu160, Phe282, Phe283, Asp351, Asp372, Asn379, Glu380, and Glu387 to Ala, respectively. Each of these mutants was coexpressed with Ceg3 and ADPR modification of ANTs was examined. None of these mutations affected the stability of Larg1 but the ability of these mutants to remove the ADPR moiety from modified ANTs has almost completely been abolished (Figure 5D). Thus, residues in Larg1 involved in engaging the ADPR moiety are important for its activity in removing the modification from ADP‐ribosylated ANTs.

### Larg1 interferes with Ceg3‐induced ADPR of ANTs during *L. pneumophila* infection

**Figure 6:**
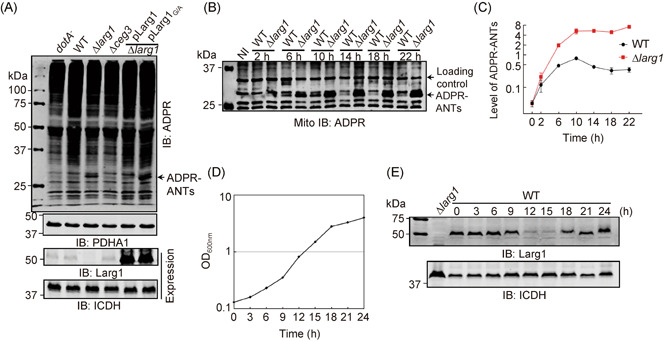
Ceg3 and Larg1 temporally regulate ADP‐ribosylation (ADPR) of ANTs in cells infected with *Legionella pneumophila*. (A) Overexpression of Larg1 but not its inactive mutant Larg1_G/A_ in the ∆*larg1* mutant abolished ADPR of ANTs in infected cells. HEK293T cells transfected to express the FcγII receptor were infected with opsonized *L. pneumophila* at an MOI of 100 for 2 h. The mitochondrial fractions were probed for ADPR modification. PDHA1 was probed as a mitochondrial marker to examine the effectiveness of the fractionation (upper two panels). The expression of Larg1 in these bacteria was detected with Larg1‐specific antibodies, with the metabolic enzyme isocitrate dehydrogenase (ICDH) being detected as a loading control (lower two panels). (B,C) ANTs modification is temporally regulated by Ceg3 and Larg1 in cells infected by *L. pneumophila*. HEK293T cells transfected to express the FcγII receptor were infected with opsonized wild‐type *L. pneumophila* or the ∆*larg1* strain at an MOI of 20 for the indicated durations. Proteins of isolated mitochondria resolved by SDS‐PAGE were probed with ADPR‐specific antibodies. The band above ADPR‐ANTs nonspecifically recognized by the antibodies was used as a loading control (B). Quantitation of modified ANTs in samples infected with wild‐type *L. pneumophila* or the ∆*larg1* mutant. The intensity of the loading control bands was used for normalization by Image Studio. Results shown were from three independent experiments (mean ± SEM) (C). (D,E) Expression of Larg1 is induced in the lag phase and stationary phase. Growth of *L. pneumophila* in AYE broth was monitored by measuring OD_600_ at the indicated time points (D); samples withdrawn from the cultures were probed with Larg1‐specific antibodies (E). The ∆*larg1* mutant grown to the post‐exponential phase was included. ICDH was detected as a loading control. ANT, ADP/ATP translocase; HA, hemagglutinin; MOI, multiplicity of infection; SDS‐PAGE, sodium dodecyl sulfate polyacrylamide gel electrophoresis.

To test whether Larg1 targets modified ANTs under physiological conditions, we examined its ability to interfere with Ceg3‐induced ADPR of ANTs in cells infected with *L. pneumophila*. Consistent with earlier results^13^, modified ANTs were undetectable in samples challenged with wild‐type *L. pneumophila* at 2 h postinfection (psi) (Figure 6A). Intriguingly, modified ANTs were detected in samples infected with the Lp02∆*larg1* mutant in the same time frame (Figure 6A, third lane). Complementation of strain Lp02∆*larg1* with a multicopy plasmid that expresses Larg1 made ANTs' modification undetectable, which did not occur in the strain expressing the G/A mutant (Figure 6A, fifth and sixth lane). These results suggest that Larg1 inhibits ADPR of ANTs in cells infected with wild‐type *L. pneumophila*.

Next, we examined the role of Larg1 in the kinetics of ANTs' ADPR in cells infected with *L. pneumophila*. We consistently observed that ADPR of ANTs in cells infected with wild‐type *L. pneumophila* did not become detectable until after the infection had proceeded for 6 h; the modification peaked at 10 h psi and began to decrease from 14 h psi (Figure 6B,C). In contrast, in cells infected with the strain lacking *larg1*, the levels of ADPR‐ANTs were much higher than those in samples infected with the wild‐type strain at all infection time points examined (Figure 6B,C). Together, these results suggest that Larg1 functions to counteract the activity of Ceg3 and such activity becomes more apparent in the first few hours and when the infection has proceeded for about 14 h.

We further examined the notion that Larg1 works with Ceg3 to temporally regulate the activity of ANTs in cells infected with *L. pneumophila* by probing its protein level at different growth phases in broth‐grown bacteria. We observed that the expression of Larg1 exhibited an on–off–on pattern: it reached the highest level at both lag (0–9 h) and the post‐exponential phases (18–24 h), and was barely detectable in bacteria grown at the exponential phase (12–15 h) (Figure 6D,E). These results suggest that Larg1 functions to modulate ANTs modification at the initial and late phases of infection, which is in line with the pattern of ANTs modification in cells infected with wild‐type *L. pneumophila* that was barely detectable prior to 6 h, readily detectable between 6 and 10 h, and becomes undetectable at and beyond 14 h (Figure 6B).

### Larg1 counteracts Ceg3‐mediated inhibition of ADP/ATP exchange activity

**Figure 7:**
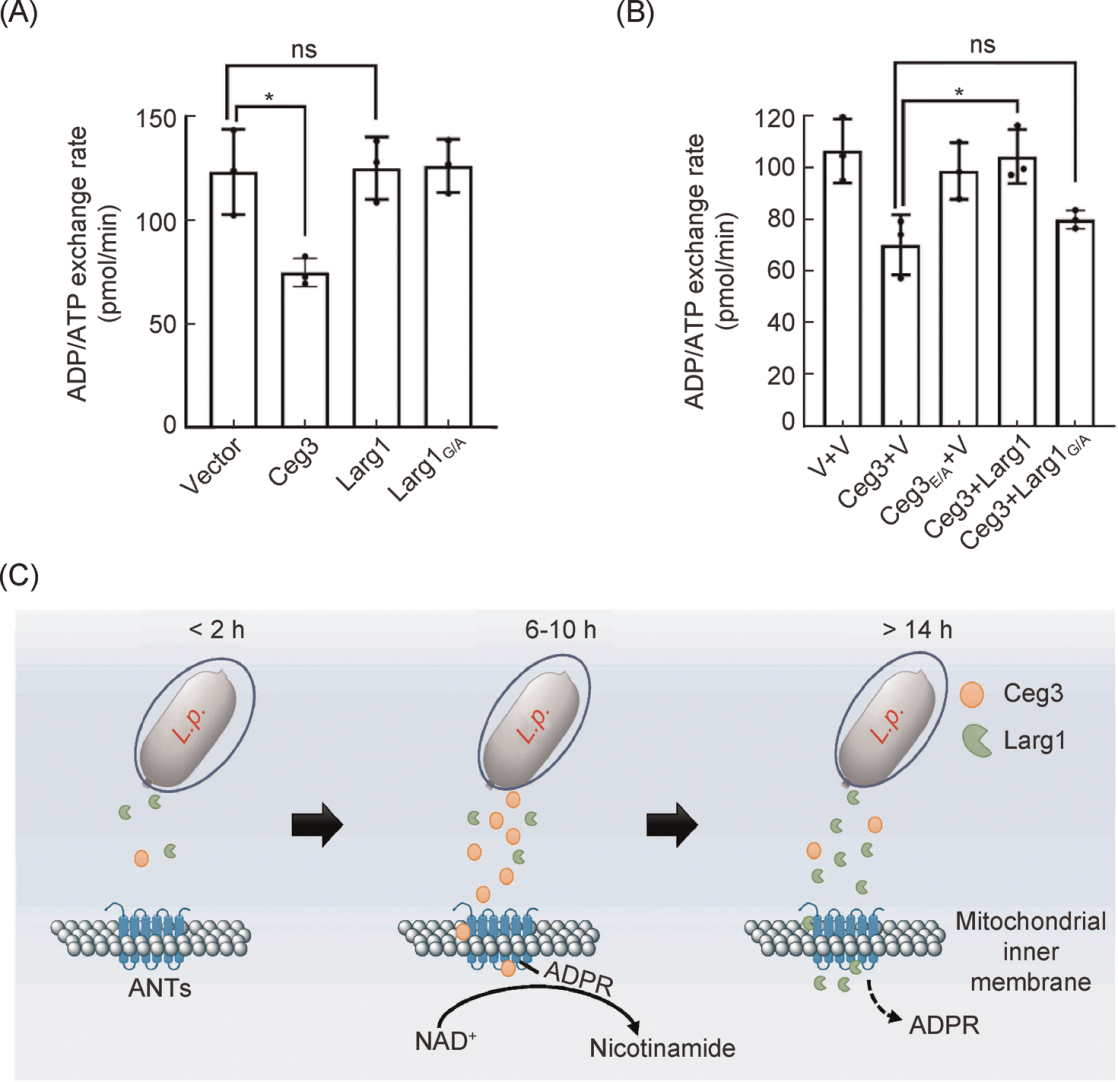
Larg1 suppresses the inhibitory effects of Ceg3 on ADP/ATP exchange by mitochondria. (A) Larg1 does not interfere with the ADP/ATP exchange by mitochondria. Mitochondria isolated from HEK293T cells transfected to express HA‐Larg1 or its inactive mutant Larg1_G/A_ were used to determine the ADP/ATP exchange rates. Results shown were from three independent experiments. Error bars: standard error of the mean (SEM). Statistical analysis was determined by a two‐tailed *t* test. ns, not significant; *p < 0.05. (B) Larg1 relieved Ceg‐3‐induced ADP/ATP exchange inhibition. Mitochondria isolated from HEK293T cells expressing the indicated proteins were processed to calculate the ADP/ATP exchange rates. Quantitation shown was from three independent experiments. Error bars: standard error of the mean (SEM). Statistical analysis was determined by a two‐tailed *t* test. ns, not significant; *p < 0.05. (C) A model for the temporal regulation of the activity of ANTs by Ceg3 and Larg1 at different stages of *Legionella pneumophila* infection. The modification of ANTs by Ceg3 did not become evident until after the infection has proceeded for 6 h, a time when the amount of Ceg3 is dominant over that of Larg1. Less or no ANT modification occurs within a few hours after bacterial uptake and at the late phase (beyond 14 h) of the infection when the abundance (activity) of Larg1 is higher than that of Ceg3.

Since ADPR of ANTs by Ceg3 leads to inhibition of ADP/ATP exchange by mitochondria. We first examined whether Larg1 itself affects the ADP/ATP exchangeability of mitochondria. Mitochondria isolated from HEK293T cells transfected to express Larg1 or its enzymatically inactive mutant Larg1_G/A_ displayed similar ADP/ATP exchangeability at levels comparable to mitochondria isolated from cells transfected with the empty vector (Figure 7A), suggesting that Larg1 itself does not affect the ADP/ATP exchange process. In contrast, the expression of Ceg3 but not Ceg3_E/A_ decreased the ADP/ATP exchange rate in mitochondria (Figure 7A,B). More importantly, such inhibition could be suppressed by coexpressing Larg1 but not its enzymatically inactive mutant Larg1_G/A_ (Figure 7B). Thus, Larg1 functions to reverse the effects of Ceg3 on ADP/ATP exchange.

## DISCUSSION

Regulation of protein activity by ADPR is one of the first PTMs known to be catalyzed by bacterial virulence factors^47^ and the number of bacterial and viral proteins that possess this activity is large^48^. Similarly, the cellular processes attacked by this PTM are also highly diverse and this has been further expanded in the past several years as exemplified by autophagy inhibition by SopF from *Salmonella typhimurium* ^49^, anti‐CRISPR protein by AcrlF11 from *Pseudomonas aeruginosa* ^50^, the modification of ubiquitin thus blocking its role in signaling by CteC from *Chromobacterium violaceum* ^51^ and ubiquitin activation for phosphoribosyl ubiquitination by members of the SidE effector family from *L. pneumophila* ^52^. Furthermore, a recent study identified an ADPR‐like modification called ADP‐riboxanation catalyzed by OspC3 from *Shigella flexneri* to block pyroptosis by inactivating caspase 11^53^. Interestingly, despite all using NAD as the reaction precursor and the modifications being chemically similar, OspC3 shows little similarity to ARTs^53^. Our study has revealed that *L. pneumophila* utilizes a pair of effectors to precisely regulate host energy production in the mitochondria by reversible ADPR.

It is well‐established that modulation of host processes by *L. pneumophila* often occurs in a balanced manner in which the activity of a virulence factor is specifically regulated by one or more effectors^54^. These regulatory effectors, also called metaeffectors, function either to fine‐tune the activity of the cognate factor or to return host targets to their original active form^19^. Our results herein indicate that Larg1 and Ceg3 work together to regulate the activity of mitochondrial ATP/ADP translocases by reversible mono ADPR. Larg1 has been suggested to be a metaeffector for Ceg3 due to the ability to suppress its yeast toxicity of the latter^19^. Our results reveal that it modulates the activity of ANTs by removing the ADPR modification induced by Ceg3 with an enzymatic activity that cleaves the N‐glycosidic bond between the modifier and the arginine residues, leading to the return of ANTs back to their original forms that are active in ADP/ATP exchange (Figure 4). Our results also suggest that the activity of ANTs in cells infected with *L. pneumophila* is temporally regulated by the Ceg3 and Larg1. In the first 6 h of infection, ADP‐ribosylated ANTs were not detectable using ADPR‐specific antibodies in experiments with wild‐type bacterium, but can be readily detected in samples infected with the ∆*larg1* mutant, suggesting that Larg1 reduces the level of ADP‐ribosylated ANTs in this time frame, which is consistent with its obvious induction in the lag phase of its growth cycle (Figure 6). Intriguingly, the level of Larg1 became barely detectable when the bacteria reach the exponential phase (12–15 h), and the level of ADP‐ribosylated ANTs starts to decline after the infection proceeds for 14 h (Figure 6). This fluctuation is consistent with the pattern of *larg1* expression in the bacterium, which is accumulated at high levels again after disappearance between 12 h and 15 h (Figure 6). Under our experimental conditions, it appears that in the first few hours after bacterial uptake, the activity of Larg1 is dominant over that of Ceg3; this balance is shifted to Larg1 at least 6 h after the infection has started as its expression begins to decline. The activity of the eraser becomes dominant again 14 h after uptake (Figure 7C). These results suggest that the expression of Ceg3 and Larg1 is independently regulated, which is consistent with the fact that these genes are transcribed convergently (Figure 1B). Yet, how the bacterium is benefited by inhibiting ANTs at a time it is undergoing active replication needs further investigation.

Differing from Ceg3, which is almost exclusively targeted at the mitochondria^13^, only a fraction of Larg1 is localized to this organelle (Figure 3). This phenomenon is not unique to this metaeffector pair. For example, SidM/DrrA involved in the regulation of the small GTPase Rab1 is specifically targeted at the LCV^5^, ^55^, but its metaeffector SidD is mostly cytosolic^28^. Similarly, the phosphorylcholine transferase AnkX largely is associated with the ER, whereas its metaeffector Lem3 does not display such preference^30^. It seems that whereas enzymes that function to impose the regulation by posttranslational modifications often have evolved to more specifically target the organelle that houses their substrates, cellular localization of enzymes that function to reverse the modification is less stringent. Mistargeting of an enzyme capable of interfering with host processes clearly will more likely to cause unintended damage, whereas the reversal of a modification can be achieved even if only a fraction of the enzyme can reach the location within which the modified substrate resides.

Macrodomain‐containing proteins involved in ADPR removal have been identified in a large number of microorganisms^56^. For example, DrPARG from *D. radiodurans* is an endo‐glycohydrolase against poly‐ADP‐ribose^38^. The viral macrodomains from positive single‐stranded (+) ssRNA viruses, such as Chikungunya virus, effectively reverse mono‐ADPR on proteins^57^. Although the conserved motif GGG‐X_6‐8_‐QEE involved in the formation of macrodomains associated with typical ADP‐ribosylhydrolases such as TcPARG^45^ and DrPARG^38^ is not present in Larg1, its catalytic loop is structurally similar to macrodomains in bacterial PARGs (Figure 5A). The configuration of its ADPR binding pocket and how relevant residues interact with the substrate suggest that Larg1 utilizes a unique macrodomain to recognize ADPR for subsequent cleavage of the N‐glycosidic bond between ADPR and ANTs (Figure 5B,C). Further structural analysis of the complexes such as Larg1‐ADPR‐ANTs will shed light on the substrate specificity of this ADP‐ribosylhydrolase.

## MATERIALS AND METHODS

### Media, bacterial strains, plasmid construction, and cell lines

*L. pneumophila* strains used in this study were constructed from Lp02, a strain derived from strain Philadelphia 1^58^. Strain Lp03 defective in the *dot/icm* system (*dotA*) has been previously described^59^. *L. pneumophila* was cultured following the standard procedure^58^ in ACES‐buffered yeast extract (AYE) broth or on charcoal yeast extract (CYE) agar plates. To maintain plasmids in *L. pneumophila*, antibiotics were used at the following concentrations: kanamycin, 20 μg/ml; streptomycin, 100 μg/ml. When needed, thymidine was added at a final concentration of 100 μg/ml. *Escherichia coli* strains were grown in Luria–Bertani (LB) broth or on LB agar plates that contain antibiotics at the following concentrations: ampicillin, 100 μg/ml; kanamycin, 30 μg/ml.

The *∆larg1* in‐frame deletion strain was constructed by a two‐step allelic exchange strategy as described^60^. To construct complementation strains, genes were inserted into the pZLQ‐Flag^25^, which will add a Flag tag to the amino end of the inserted gene. For ectopic expression of proteins in mammalian cells, genes were cloned into the pFlag‐CMV vector^61^ or pAPH‐HA^62^, which produces Flag‐ or HA‐tagged proteins. The integrity of all constructs was verified by sequencing analysis. HEK293T or Raw264.7 cells purchased from the ATCC were cultured in Dulbecco's modified minimal Eagle's medium or Roswell Park Memorial Institute 1640 medium supplemented with 10% fetal bovine serum. All mammalian cell lines were regularly checked for potential mycoplasma contamination by the universal mycoplasma detection kit from ATCC (cat# 30‐1012 K). The sequences of primers, plasmids, and bacterial strains used in this study are listed in Table S1.

### Yeast toxicity assays

The yeast strain W303^16^ was used to constructed all strains described in this study; yeast was grown at 30°C in a YPD medium or in synthetic media with appropriate amino acid combinations and 2% of glucose or galactose as the sole carbon source. Yeast transformation was performed as previously described^63^. For the suppression of Ceg3 yeast toxicity, *ceg3* was inserted into pSB157m^61^ and transformed into W303 to make W303(pSB157::Ceg3), which was used to test the suppression activity of Larg1 expressed from p424TEF^64^. Yeast grown in appropriate liquid dropout media supplemented with glucose was washed and serially diluted in sterile water and 5 μl of each dilution was spotted onto selective plates containing glucose or galactose. Images of the plates were acquired after incubation at 30°C for 2 days.

### Transfection, immunoprecipitation, and bacterial infection

Lipofectamine 3000 (cat# L3000150; Invitrogen) was used to transfect HEK293T cells with relevant plasmids. Cells were collected and lysed with the Tris‐buffered saline buffer (150 mM NaCl, 50 mM Tris‐HCl, pH 7.5) containing 1% Triton X‐100 24 h after transfection. To purify ADPR Flag‐ANT1, immunoprecipitation was performed with lysates of cells coexpressing HA‐Ceg3 and Flag‐ANT1 by using beads coated with the Flag antibody (cat# F2426; Sigma‐Aldrich) at 4°C overnight. Beads were washed three times with prechilled lysis buffer. Flag‐ANT1 was eluted from beads using a Flag peptide solution (150 μg/ml) (cat# F3290; Sigma‐Aldrich).

For infection experiments with *L. pneumophila*, we grew bacterial strains to the postexponential phase in AYE broth (OD_600_ = 3.2–4.0) and checked their motility by a light microscope. When needed, complementation strains in which the genes of interest were under control of the P_tac_ promoter were induced with 0.2 mM isopropyl β‐d‐1‐thiogalactopyranoside (IPTG) for 4 h at 37°C before infection. To determine the modification of ANTs in cells infected with relevant *L. pneumophila* strains, we first transfected HEK293T cells to express the FCγRII receptor for 24 h, and the cells were then infected with bacteria opsonized by rabbit antibodies specific for strain Lp02^52^. Mitochondria were isolated from infected cells using a kit (cat# 89874; Thermo Fisher Scientific), as per the manufacturer's instructions. To evaluate intracellular growth of *L. pneumophila*, we infected approximately 4 × 10^5^ Raw264.7 cells or *D. discoideum* seeded in 24‐well plates at a multiplicity of infection (MOI) of 0.05.  2 h after adding the bacteria, infections were synchronized by washing the cells three times with warm PBS buffer and the samples were incubated at 37°C or 25°C, respectively. At the indicated time points, cells were treated with 0.02% saponin, dilutions of the cell lysates were plated onto CYE plates, and the bacteria counts were determined by enumerating CFUs from triplicate samples of each strain.

### Protein purification

To produce His_6_‐tagged recombinant protein, 20 ml overnight *E. coli* cultures were transferred to 400 ml LB medium supplemented with 100 μg/ml ampicillin and grown to OD_600nm_ of 0.6–0.8 at 37°C. Protein expression was induced with 0.2 mM IPTG at 18°C for 16–18 h on a shaker (250 rpm). Bacterial cells were collected by centrifugation and lysed by sonication. The soluble lysates were cleared by spinning at 12,000*g* at 4°C. His_6_‐tagged proteins were purified with Ni^2+^‐NTA beads (Qiagen) and were eluted with PBS buffer containing 300 mM imidazole. Purified proteins were dialyzed against PBS buffer containing 5% glycerol and 1 mM dithiothreitol (DTT) at 4°C for 14 h.

To purify proteins for structural study, the coding region of *lpg0081* (16–428aa) was inserted into pET28a‐sumo and the resulting plasmid was transformed into *E. coli* BL21(DE3) and B834 for expression of native and Se‐Met proteins, respectively. The bacterial strains were cultured at 37°C in LB on a shaker (220 rpm) and then induced with 0.5 mM IPTG when the bacteria grew to a density OD_600_ = 0.8. The bacteria were then cultured for 16 h at 18°C before collecting the cells by centrifugation (5000*g*, 15 min). Cells resuspended in a cold lysis buffer (50 mM Tris–HCl pH 7.5, 150 mM NaCl) were lysed by ultrasonication. Lysates were centrifuged at 35,000*g* for 30 min at 4°C to obtain supernatant, which was used to purify His_6_‐tagged proteins by affinity chromatography (Ni^2+^ resin). The SUMO tag was removed by the SUMO protease ULP1. Proteins were further purified by size‐exclusion chromatography using a Superdex 200 Increase column (GE Healthcare) equilibrated with a buffer containing 25 mM HEPES, pH 7.5, 150 mM NaCl, and 2 mM DTT. Collected proteins were concentrated to *A*_280_ = 10 for crystallization screen.

### Antibodies and immunoblotting

Purified His_6_‐Larg1 was used to raise mouse‐specific antibodies using a standard protocol (Pocono Rabbit Farm & Laboratory). The antibodies were affinity‐purified as previously described^65^. For immunoblotting, samples resolved by SDS‐PAGE were transferred onto 0.2 μm nitrocellulose membranes (cat# 1620112; Bio‐Rad), which were blocked with 5% nonfat milk at room temperature for 1 h before being incubated with the appropriate primary antibodies: anti‐Flag (cat# F1804; Sigma‐Aldrich), 1: 5000; anti‐HA (cat# H3663; Sigma‐Aldrich), 1:5000; anti‐tubulin (E7; DSHB) 1:10,000; anti‐ADPR (cat# MABE1016; Sigma‐Aldrich), 1:1000; anti‐ICDH^66^, 1:10,000; anti‐PDHA1 (cat# 18068‐1‐AP; Proteintech), 1:5000; anti‐ATPB (cat# 17247‐1‐AP; Proteintech), 1:2000; anti‐TOM20 (cat# 11802‐1‐AP; Proteintech), 1:5000; anti‐VDAC1 (cat# 55259‐1‐AP; Proteintech), 1:2000; anti‐Cyto‐c (cat# sc‐13560; Santa Cruz), 1:1000; anti‐Larg1, 1:2500. Membranes were then incubated with appropriate IRDye infrared secondary antibodies and scanned by an Odyssey infrared imaging system (Li‐Cor's Biosciences).

### Immunostaining

HeLa cells seeded at 5 × 10^4^ per well on glass coverslips in 24‐well plates for 24 h were transfected with the relevant plasmids. Eighteen hours after transfection, the culture medium was replenished with a fresh medium containing 500 nM Mitotracker Red (cat# M22425; Thermo Fisher Scientific). After incubation at 37°C for 30 min, cells were fixed with 4% formaldehyde for 30 min at 4°C and were permeabilized using 0.2% Triton X‐100 (5 min). After blocked with 4% goat serum for 30 min at 37°C, HA was stained with the HA‐specific antibody (cat# H3663; Sigma‐Aldrich) at a 1:50 dilution for 15 h at 4°C. Washed samples were then stained with secondary antibodies conjugated to Alexa Flour 594 or Alexa Flour 488 (Thermo Fisher Scientific) at a dilution of 1:500 for 1 h at room temperature. For colocalization with ANT1, mCherry‐ANT1 was cotransfected with HA‐Larg1, and the latter was similarly stained. In each case, the images were taken using a Zeiss LSM 880 confocal microscope after nucleus staining with Hoechst.

### ADP/ATP exchange rate determination

We isolated mitochondria from HEK293T cells transfected to express the proteins of interest, the samples were resuspended in a reaction buffer (10 mM HEPES [pH 7.4], 250 mM sucrose, and 10 mM KCl). The ADP/ATP exchange process was initiated by the addition of 2 mM ADP. After 5 min of incubation, the amount of ATP in the supernatant was determined using a kit for ATP measurement (cat# A22066; Invitrogen). Luminescence was detected with a BioTek plate reader (Synergy 2; BioTek). The readings were converted into concentrations using a standard curve prepared with serially diluted ATP for each experiment.

### LC‐MS/MS analysis

Protein bands were digested in‐gel with Lys‐C as previously described^67^. Digested peptides were analyzed by LC‐ESI‐MS/MS using the Dionex UltiMate 3000 RSLC nano System coupled to the Q Exactive™ HF Hybrid Quadrupole‐Orbitrap Mass Spectrometer (Thermo Fisher Scientific) as described previously^68^, ^69^. Reverse‐phase peptide separation was performed using a trap column (300 μm ID × 5 mm) packed with 5 μm 100 Å PepMap C18 medium, and then separated on a reverse‐phase column (50 cm long × 75 µm ID) packed with 2 µm 100 Å PepMap C18 silica (Thermo Fisher Scientific). The temperature of the column was maintained at 50°C.

Mobile phase solvent A was 0.1% FA in water and solvent B was 0.1% FA in 80% ACN. Loading buffer was 98% water/2% ACN/0.1% FA. Peptides were separated by loading into the trap column in a loading buffer for 5 min at 5 µl/min flow rate and eluted from the analytical column at a flow rate of 150 nl/min using a 130 min LC gradient as follows: linear gradient of 5%–27% of solvent B in 80 min, 27%–45% in next 20 min, 45%–100% of B in next 5 min at which point the gradient was held at 100% of B for 7 min before reverting back to 2% of B at 112 min, and holding at 2% of B for next 18 min for equilibration. The MS was operated in positive ion and standard data‐dependent acquisition mode with Advanced Peak Detection function activated for the top 20n. The fragmentation of precursor ion was accomplished by a stepped normalized collision energy setting of 27%. The resolution of the Orbitrap mass analyzer was set to 120,000 and 15,000 for MS1 and MS2, respectively. The full scan MS1 spectra were collected in the mass range of 350–1600 *m*/*z*, with an isolation window of 1.2 *m*/*z* and a fixed first mass of 100 *m*/*z* for MS2. The spray voltage was set at 2 and Automatic Gain Control target of 4e5 for MS1 and 5e4 for MS2, respectively. Peptides of interest were further analyzed in Xcalibur QualBrowser.

### Crystallization, data collection, and structural determination

The Larg1‐ADPR complex was prepared by incubating Larg1 and ADPR with a molar ratio of 1:3 for 1 h on ice. Crystallization screens were conducted using the hanging‐drop vapor diffusion method at 16°C, with drops containing 0.5 µl of the protein solution mixed with 0.5 µl of reservoir solution. Diffraction quality of the Larg1‐ADPR complex crystals was obtained in 0.2 M lithium sulfate, 0.1 M Tris, pH 7.5, and 5% polyethylene glycol 4000, respectively. Crystals were harvested and flash‐frozen in liquid nitrogen with 20% glycerol as a cryoprotectant. Complete X‐ray diffraction data sets were collected at BL02U1 beamline of Shanghai Synchrotron Radiation Facility (SSRF). Diffraction images were processed with the HKL‐2000 program^70^. The initial phase of Larg1 was determined by using the single‐wavelength anomalous dispersion phasing method. Phases were calculated using AutoSol implemented in PHENIX. AutoBuild in PHENIX was used to automatically build the atom model. Molecular Replacement was then performed with this model as a template to determine the structure of the Larg1‐ADPR complex (PDB:7W3S). Model building and crystallographic refinement were carried out in Coot and PHENIX^71^, ^72^. Detailed data collection and refinement statistics are listed in Table 1. The interactions were analyzed with PyMOL (http://www.pymol.org/) and PDBsum and figures were generated with PyMOL.

**Table 1:** X‐ray crystallography data collection and refinement statistics.

Data set	Larg1‐apo (Se‐Met)	Larg1‐ADPR complex
**Data collection**		
Beamline	BL02U1, SSRF	BL02U1, SSRF
Wavelength (Å)	0.97918	0.97918
Resolution range^a^^,^ b	28.71–2.44 (2.527–2.44)	53.42–2.324 (2.407–2.324)
Space group	C 1 2 1	P 1 21 1
**Cell dimensions**		
a,b,c (Å)	119.09, 93.25, 108.75	109.25, 92.81, 116.05
α, β, γ (°)	90.00, 106.48, 90.00	90.00,105.33,90.00
Total reflections	84419 (8431)	187616 (18025)
Unique reflections	42430 (4225)	94202 (9251)
Multiplicity	2.0 (2.0)	2.0 (1.9)
Completeness (%)	99.63 (99.76)	97.54 (97.05)
Mean I/sigma (I)	11.43 (1.83)	12.09 (2.26)
*R*‐merge	0.055 (0.4794)	0.03957 (0.3461)
*R*‐meas	0.07778 (0.678)	0.05595 (0.4894)
*R*‐pim	0.055 (0.4794)	0.03957 (0.3461)
CC1/2	0.995 (0.759)	0.998 (0.773)
**Refinement**		
Reflections used in refinement		94051 (9250)
Reflections used for *R*‐free		4598 (445)
*R*_work_		0.1860 (0.2640)
*R*_free_		0.2270 (0.3291)
Wilson B‐factor (Å^2^)		36.30
Number of nonhydrogen atoms		14040
Macromolecules		13191
Protein residues		1652
RMS (bonds)		0.008
RMS (angles)		0.97
Ramachandran favored (%)		95.28
Ramachandran allowed (%)		4.41
Ramachandran outliers (%)		0.31
Rotamer outliers (%)		0.56
Clash score		7.72
Average B‐factor (Å^2^)		42.29

^a^ For each structure, one crystal was used. ^b^Values in parentheses are for the highest‐resolution shell. ADPR, ADP‐ribosylation; RMS, root mean square; SSRF, Shanghai Synchrotron Radiation Facility.

### Isothermal titration calorimetry

ITC experiments were carried out using a Low Volume Nano ITC (TA instruments) set at 16°C. The proteins and ADPR were dissolved in the buffer containing 50 mM Tris‐HCl, pH 7.5, and 150 mM NaCl. The concentration of ADPR in the syringe was 1 mM and the concentrations of proteins in the sample cell were 0.1 mM. Twenty‐five consecutive 2 µl injections of ADPR were titrated into a 350 µl sample cell with a 200 s interval between injections using a stirring rate of 120 rpm. A single‐site binding model was used for nonlinear curve fitting using the Launch NanoAnalyze software provided by the manufacturer. ITC titration was repeated at least twice for each experiment.

### Data quantitation and statistical analyses

Student's *t* test was used to compare the mean levels between two groups each with at least three biological repeat samples.

## AUTHOR CONTRIBUTIONS

Jiaqi Fu and Zhao‐Qing Luo conceived the project; Jiaqi Fu, Dan Huang, and Lei Song performed research; Jiaqi Fu and Zhao‐Qing Luo analyzed the results; Pengwei Li and Hongxin Guan determined the structures; Pengwei Li, Hongxin Guan, and Songying Ouyang analyzed structural results; Lei Song provided materials and resources; Jiaqi Fu wrote the first draft of the paper; Jiaqi Fu and Zhao‐Qing Luo revised the manuscript with input from all authors.

## ETHICS STATEMENT

This study did not use animals.

## CONFLICT OF INTERESTS

The authors declare no conflict of interests.

## Supporting information


**S**upporting information.

Supporting information.

## Data Availability

All data described have been included in the paper; the PDB entry of the structure of the Larg1‐ADPR complex is: 7W3S.
