# Supplementary figures and images for: Legionella pneumophila temporally regulates the activity of ADP/ATP translocases by reversible ADP‐ribosylation
Source: mLife. 2022 Mar 30;1(1):51–65. doi: 10.1002/mlf2.12014 (PMC10989772; doi:10.1002/mlf2.12014)

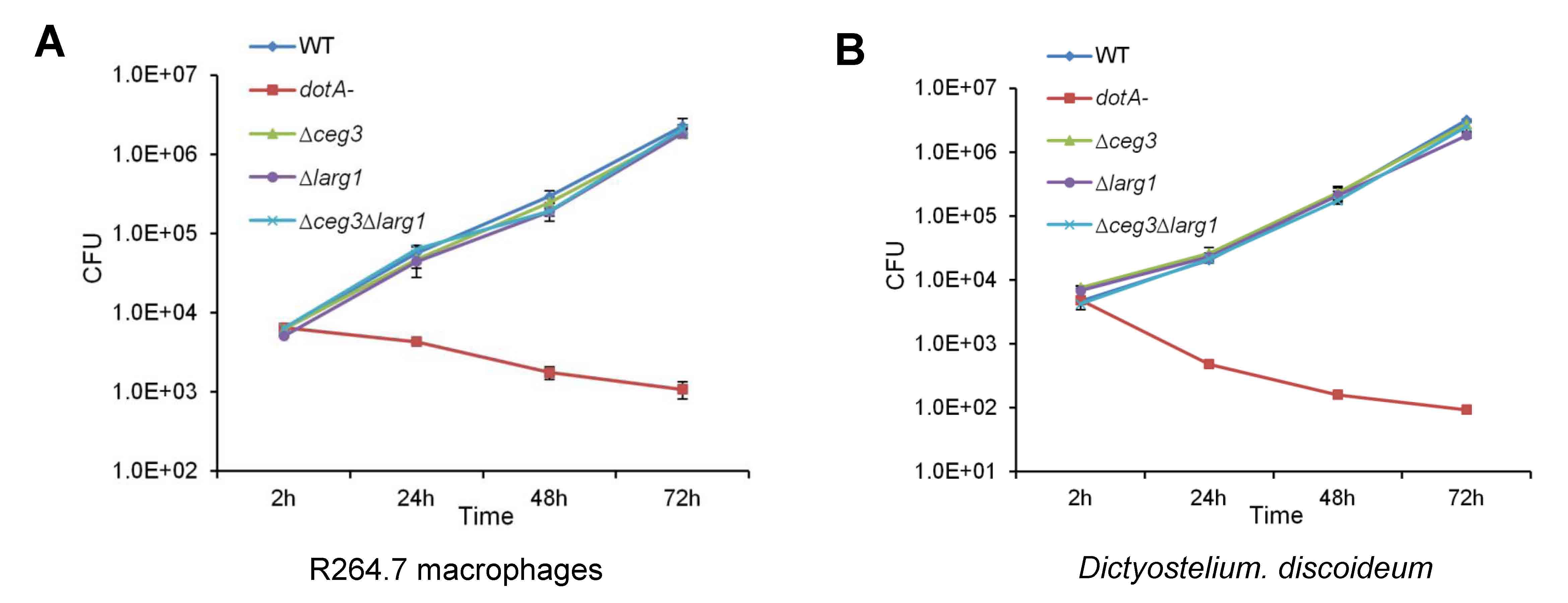

Supplement: Supplementary file 1 — Supporting information. [file MLF2-1-51-s002.tif]
