# Supplementary material for: Legionella pneumophila temporally regulates the activity of ADP/ATP translocases by reversible ADP‐ribosylation
Source: mLife. 2022 Mar 30;1(1):51–65. doi: 10.1002/mlf2.12014 (PMC10989772; doi:10.1002/mlf2.12014)
Supplement: Supplementary file 2 — Supporting information. [file MLF2-1-51-s001.docx]

Table S1 Bacterial strains, plasmids and primers used in this study

| Bacterial Strains | Source | Identifier |
| --- | --- | --- |
| *L. pneumophila* (Philadelphia-1) Lp02 | (Berger and Isberg, 1993) | N/A |
| *L. pneumophila* Lp03 | (Berger and Isberg, 1993) | N/A |
| Lp02Δ*larg1* | This study | N/A |
| Lp02Δ*ceg3* | This study | N/A |
| Lp02Δ*larg1* (pZLQ-Flag) | This study | N/A |
| Lp02Δ*larg1* (pLarg1) | This study | N/A |
| Lp02Δ*larg1* (pLarg1_G279A/G281A_) | This study | N/A |
| *E.coli* BL21(DE3) | NEB | CAT#C2527I |
| *E.coli* XL1-Blue | Agilent | CAT#200249 |
|  |  |  |

| Plasmids | Source | Identifier |
| --- | --- | --- |
| pZLQ-Flag | (Xu et al., 2010) | N/A |
| pZLQ-Falg::*larg1* | This study | N/A |
| pZLQ-Flag::*larg1_G279A/G281A_* | This study | N/A |
| pAPH-HA | This study | N/A |
| pAPH-HA::*ceg3* | This study | N/A |
| pAPH-HA::*ceg3_E141A/E143A_* | This study | N/A |
| pAPH-HA::*larg1* | This study | N/A |
| pAPH-HA::*larg1_G279A/G281A_* | This study | N/A |
| pFlagCMV::ANT1_V227K/R237K_ | This study | N/A |
| pQE30 | Qiagen | CAT#32915 |
| pQE30::*larg1* | This study | N/A |
| pQE30::*larg1_G279A/G281A_* | This study | N/A |
| pAPH-HA::*larg1_Y134A_* | This study | N/A |
| pAPH-HA::*larg1_E160A_* | This study | N/A |
| pAPH-HA::*larg1_F282A_* | This study | N/A |
| pAPH-HA::*larg1_F283A_* | This study | N/A |
| pAPH-HA::*larg1_D351A_* | This study | N/A |
| pAPH-HA::*larg1_D372A_* | This study | N/A |
| pAPH-HA::*larg1_N379A_* | This study | N/A |
| pAPH-HA::*larg1_E380A_* | This study | N/A |
| pAPH-HA::*larg1_E387A_* | This study | N/A |
| pSB157 | This study | N/A |
| pSB157::*ceg3* | This study | N/A |
| p424TEF::*larg1* | This study | N/A |
| p424TEF::*larg1_G279A/G281A_* | This study | N/A |

| Primers | Sequence (Restriction enzyme sites are underlined) | Note |
| --- | --- | --- |
| pSL1001 | ctaggatccatgcggtctaaatttttttcatttttc | *larg1* 5F BamHI |
| pSL1002 | catgtcgacctagagtttaatgctcgatgag | *larg1* 3R SalI |
| pSL1003 | catgtcgacggacaagagtcaactttaga | *larg1* up SalI  knockout |
| pSL1004 | Ctaggatcccggtcttgatgcacgtgt | *larg1* up BamHI knockout |
| pSL1005 | Ctaggatccacagtcaatccaaggcca | *larg1* down BamHI knockout |
| pSL1006 | catgagctcaggttgcctatcattcctc | *larg1* down SacI knockout |
| pSL1007 | ttaaagccactgcggttgcaatggctttttttgcaaaaattgat | *larg1_G279A/G281A_* -1 |
| pSL1008 | atcaatttttgcaaaaaaagccattgcaaccgcagtggctttaa | *larg1_G279A/G281A_* -2 |
| pSL1009 | cgcctccggtggtgctccactaaccaat | *larg1_Y134A_* -1 |
| pSL1010 | attggttagtggagcaccaccggaggcg | *larg1_Y134A_* -2 |
| pSL1011 | aggcccctcttttgcaaataattacctcca | *larg1_E160A_* -1 |
| pSL1012 | tggaggtaattatttgcaaaagaggggcct | *larg1_E160A_* -2 |
| pSL1013 | ctgcggttggaatgggtgcttttgcaaaaat | *larg1_F282A_* -1 |
| pSL1014 | atttttgcaaaagcacccattccaaccgcag | *larg1_F282A_* -2 |
| pSL1015 | ggttggaatgggttttgctgcaaaaattgattgc | *larg1_F283A_* -1 |
| pSL1016 | gcaatcaatttttgcagcaaaacccattccaacc | *larg1_F283A_* -2 |
| pSL1017 | gaagcacacgcgctgttctggagtttc | *larg1_D351A_* -1 |
| pSL1018 | gaaactccagaacagcgcgtgtgcttc | *larg1_D351A_* -2 |
| pSL1019 | ctattaatccttcagctgcgtttgctctaac | *larg1_D372A_* -1 |
| pSL1020 | gttagagcaaacgcagctgaaggattaatag | *larg1_D372A_* -2 |
| pSL1021 | gtttgctctaactggagctgaatggggatatgg | *larg1_N379A_* -1 |
| pSL1022 | ccatatccccattcagctccagttagagcaaac | *larg1_N379A_* -2 |
| pSL1023 | ctaactggaaatgcatggggatatggt | *larg1_E380A_* -1 |
| pSL1024 | accatatccccatgcatttccagttag | *larg1_E380A_* -2 |
| pSL1025 | atatggtagcgttgcatcaatgattggga | *larg1_E387A_* -1 |
| pSL1026 | tcccaatcattgatgcaacgctaccatat | *larg1_E387A_* -2 |
